# Feasibility of the Web-Based Intervention Designed to Educate and Improve Adherence Through Learning to Use Continuous Glucose Monitor (IDEAL CGM) Training and Follow-Up Support Intervention: Randomized Controlled Pilot Study

**DOI:** 10.2196/15410

**Published:** 2021-02-09

**Authors:** Madison B Smith, Anastasia Albanese-O'Neill, Yingwei Yao, Diana J Wilkie, Michael J Haller, Gail M Keenan

**Affiliations:** 1 College of Nursing University of Florida Gainesville, FL United States; 2 Department of Pediatrics College of Medicine University of Florida Gainesville, FL United States; 3 Department of Biobehavioral Nursing Science College of Nursing University of Florida Gainesville, FL United States; 4 Department of Family, Community and Health Systems Science College of Nursing University of Florida Gainesville, FL United States

**Keywords:** type 1 diabetes mellitus, continuous glucose monitor, web-based training, diabetes education, intervention

## Abstract

**Background:**

Proper training and follow-up for patients new to continuous glucose monitor (CGM) use are required to maintain adherence and achieve diabetes-related outcomes. However, CGM training is hampered by the lack of evidence-based standards and poor reimbursement. We hypothesized that web-based CGM training and education would be effective and could be provided with minimal burden to the health care team.

**Objective:**

The aim of this study was to perform a pilot feasibility study testing a theory-driven, web-based intervention designed to provide extended training and follow-up support to adolescents and young adults newly implementing CGM and to describe CGM adherence, glycemic control, and CGM-specific psychosocial measures before and after the intervention.

**Methods:**

The “Intervention Designed to Educate and improve Adherence through Learning to use CGM (IDEAL CGM)” web-based training intervention was based on supporting literature and theoretical concepts adapted from the health belief model and social cognitive theory. Patients new to CGM, who were aged 15-24 years with type 1 diabetes for more than 6 months were recruited from within a public university’s endocrinology clinic. Participants were randomized to enhanced standard care or enhanced standard care plus the IDEAL CGM intervention using a 1:3 randomization scheme. Hemoglobin A_1c_ levels and psychosocial measures were assessed at baseline and 3 months after start of the intervention.

**Results:**

Ten eligible subjects were approached for recruitment and 8 were randomized. Within the IDEAL CGM group, 4 of the 6 participants received exposure to the web-based training. Half of the participants completed at least 5 of the 7 modules; however, dosage of the intervention and level of engagement varied widely among the participants. This study provided proof of concept for use of a web-based intervention to deliver follow-up CGM training and support. However, revisions to the intervention are needed in order to improve engagement and determine feasibility.

**Conclusions:**

This pilot study underscores the importance of continued research efforts to optimize the use of web-based intervention tools for their potential to improve adherence and glycemic control and the psychosocial impact of the use of diabetes technologies without adding significant burden to the health care team. Enhancements should be made to the intervention to increase engagement, maximize responsiveness, and ensure attainment of the skills necessary to achieve consistent use and improvements in glycemic control prior to the design of a larger well-powered clinical trial to establish feasibility.

**Trial Registration:**

ClinicalTrials.gov NCT03367351, https://clinicaltrials.gov/ct2/show/NCT03367351.

## Introduction

### Background

Historically, adolescents and young adults have demonstrated the poorest glycemic control compared to younger children and older adults; yet, they remain the most resistant to adopting newly developed technologies that could significantly improve type 1 diabetes (T1D) outcomes [1]. The continuous glucose monitor (CGM) can substantially improve glycemic control when worn consistently [2-4]. Despite the recognized benefit, only 24% of the adolescents and 22% of the young adults with T1D are current CGM users compared to 51% and 37% of children (aged less than 6 years and 6-12 years, respectively) and 37% and 34% of the adults (aged 26-50 years and older than 50 years, respectively) [1]. Even fewer adolescents and young adults wear the device with the consistency associated with improved glycemic control [3,5]. To foster adherence to the device and improve outcomes, experts cite the importance of training and follow-up support during the first few months to ensure proper use of CGMs [6]. Thus, a pilot randomized controlled trial was implemented to evaluate the feasibility of the web-based “Intervention Designed to Educate and improve Adherence through Learning to use CGM” or the IDEAL CGM.

### CGM Use

An international consensus statement released by key leaders regarding the use of CGM in children and adolescents stated that proper training is necessary for patients to use CGM correctly [6]. Recommendations include maintaining a high level of contact with families during the first few months of wear, which incorporates start-up training and realistic expectation setting, in addition to follow-up visits after CGM implementation to download data, review alarm settings, encourage ongoing CGM use, and address potential barriers to use [6]. These efforts take a significant amount of time and health care resources without financial reimbursement available to offset costs [7]. CGM education does not yet have established standards that are widely recognized and there is little evidence available to link educational efforts to diabetes-related outcomes [7-9].

The study of human factors works to leverage the characteristics and limitations of human interactions to improve the design of systems and use of technology [10]. Psychosocial factors play a significant role in patient acceptance and use of these technologies [11]. These factors include satisfaction (hassles and benefits of use) [12-15], self-efficacy [16], quality of life [13,17,18], and emotional distress [12]. Interventions targeting human factors related to CGM use represent an opportunity to improve adherence rates and patient-reported outcomes [12]. The association between human factors and consistent use suggests that clinical interventions targeting these modifiable factors could have an effect on CGM; however, such interventions have yet to be studied [11].

### Study Intervention Rationale

Patients desire access to diabetes care that is flexible and adaptive to their individual needs in regard to timing, frequency, and form of contact [19], especially when knowledge deficiencies arise [20]. Over 96% of the young adults have been reported to seek further diabetes education outside of clinic with 81% referring to websites and 30% using web-based chat rooms and blogs [20]. The widespread acceptance of web-based resources by this population supports the use of mobile-based and web-based programs to provide tailored education to adolescents and young adult patients with T1D [21-28], without increasing the health care burden related to increased training and follow-up needs. This pilot study aimed to evaluate the feasibility of delivering a theory-driven, web-based intervention to provide follow-up training and peer support to adolescents and young adults new to CGM and to describe diabetes-related outcomes before and after the interventional period.

## Methods

### Design and Setting

Using a randomized control-group pretest-posttest design, we recruited 8 participants from a large public university’s pediatric endocrinology clinic between March 2018 and July 2018 during routine office visits and scheduled CGM trainings in clinic. Participants were randomized to enhanced standard care or enhanced standard care plus the intervention by using a 1:3 allocation scheme. This study was approved as expedited minimal risk by the University of Florida Institutional Review Board.

### Subjects

The inclusion criteria were as follows: (1) ability to read and speak English; (2) diagnosed with T1D for at least 6 months; (3) aged between 15 and 24 years at the time of enrollment; (4) access to a smartphone, tablet, or laptop/desktop computer with high speed internet access and speaker; and (5) intended use of a Dexcom G5 CGM. Participants were required to be new to CGM or have no previous CGM use within the last 3 months. Participants with significant learning disabilities or inability to comply with the study protocol were excluded. Eligible subjects were identified via a review of upcoming medical appointments, which indicated patients scheduled for CGM training. Recruitment of subjects occurred on a rolling basis within the clinical setting.

### Procedure

All participants received at least one 60-minute, face-to-face, basic CGM education and training session conducted by the regular clinical team. This training was considered enhanced standard care and took place outside of the study, prior to recruitment and enrollment (Table 1). After obtaining consent and assent (for participants aged 17 years or younger), baseline hemoglobin A_1C_ (HbA_1c_) measures were collected. A 1-week CGM run-in period was completed prior to baseline questionnaires. The web-based training intervention was delivered over a 6-week period. Adherence and glycemic control outcomes were assessed at 3 months from the baseline.

Allocation to the intervention took place using sealed envelopes generated by the investigators to reveal randomization status. Participants within the enhanced standard care group followed an identical study activity timeline, with the exception of exposure to the IDEAL CGM web-based training program. No participant was restricted from accessing additional CGM educational materials or device support throughout the study. Participants were compensated up to US $50 for completion of the initial and follow-up surveys and HbA_1c_ measures; compensation was not dependent on completion of the intervention or adherence to CGM.

**Table 1:** Study activity timeline demonstrating activities over the 3-month study period.

Activity	Week –1	Week 0	Weeks 1-6	Week 7	Weeks 11-14
Enhanced standard CGM^a^ training^b^	✓				
Study recruitment	✓				
Demographics	✓				
Surveys/tools^c^		✓		✓	
Introduction module^d^		✓			
Web-based intervention^d^			✓		
Exit satisfaction survey				✓	
Hemoglobin A_1c_ measures	✓				✓
Download CGM data^e^					✓

^a^CGM: continuous glucose monitor.

^b^Standardized training completed per clinic’s enhanced standard care, prior to enrollment in study.

^c^Includes continuous glucose monitor self-efficacy survey, satisfaction scale surveys, and knowledge assessment tool.

^d^Indicates activity only designated for the intervention arm.

^e^Objective measure of continuous glucose monitor adherence over the 3-month study period.

### IDEAL CGM Web-Based Intervention

**Figure 1:**
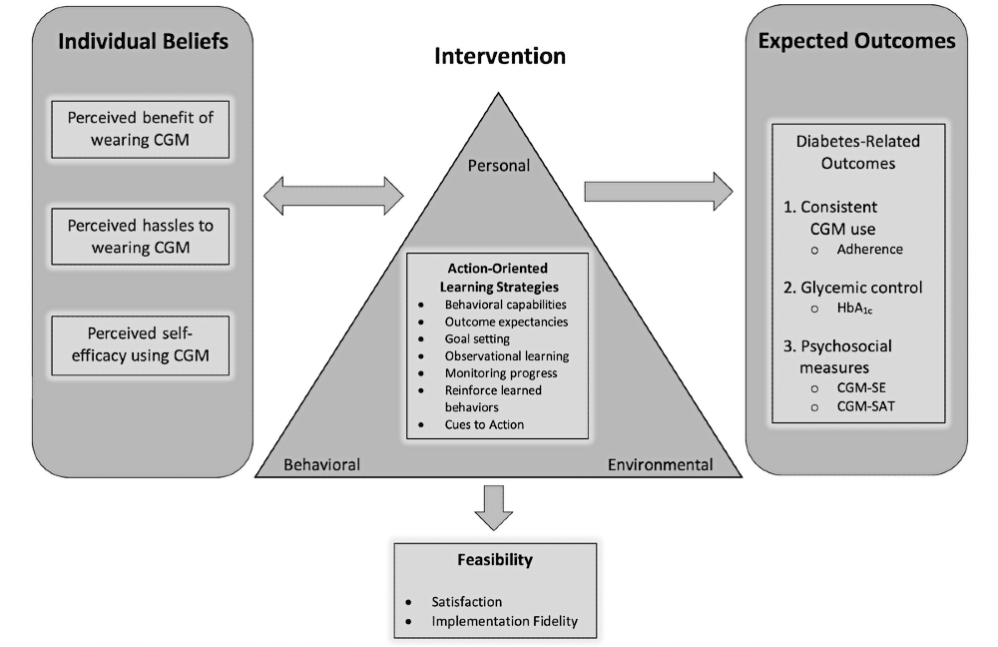
A conceptual model to support the design of the intervention and determined outcome measures. CGM: continuous glucose monitor; HbA_1c_: hemoglobin A_1c_; CGM-SE: CGM self-efficacy; CGM-SAT: CGM-satisfaction scale.

Human factors or individual beliefs associated with adherence to CGM (ie, benefits, hassles, self-efficacy) [11] are well known concepts supported by the health belief model and social cognitive theory [29,30]. The model, shown in Figure 1, used constructs of behavior change and learning theories to provide follow-up CGM training and social support to overcome perceived hassles related to CGM use and encourage behaviors that influence expected outcomes. Further, action-oriented learning strategies, seen in Table 2 [31-42], were incorporated into the IDEAL CGM intervention to create a dynamic learning process that motivated participation and skill attainment.

**Table 2:** Evidence to support action-oriented learning strategies incorporated into the web-based intervention design.

Action-oriented learning strategy	Component of intervention	Literature to support
Goal setting	Personal goal setting	1 of the 3 main factors to affect likelihood a person will change a health behavior [31]
Outcome expectancies: result an individual anticipates from taking action [31]	CGM^a^ expectation setting	1 of the 3 main factors to affect likelihood a person will change a health behavior [31]. Failure to meet expectations is one of the top cited reasons for poor CGM adherence [12,15,32-36]. Realistic expectations while using CGM were associated with better glycemic control and patient success [37]
Behavioral capabilities: knowledge and skill to perform given behavior [31]	Knowledge acquisition through provided materials	Proper training is necessary for patients to use CGM correctly [6]. Difficult to use technology is one of the top cited reasons for poor CGM adherence [12,15,32-36]
Cues to action: factors that promote action [31]	Push notifications and email reminders to access LMS^b^	Reminders to access and utilize web-based programs were critical to previously tested web-based intervention’s success [22,26,38,39]
Monitoring progress [31]. Reinforcing learned behaviors [31]	Knowledge assessment checks	Patients who consistently applied themselves to homework assignments, worksheets, and brief quizzes to reinforce learning and evaluate information gaps were observed to be most successful with SAP^c^ [9]
Observational learning (modeling): learning through the experience of credible others rather than through their own experiences [31]	Discussion boards with peers (content monitored by health care professionals)	Discussion boards were highly utilized when incorporated into program designs [22,40]. Young adults utilize web-based resources, websites, discussion boards, and blogs to augment peer and family support [41,42]. Peer-led education provided an opportunity to learn real-life explanations for problems not addressed in clinic-based learning [20]

^a^CGM: continuous glucose monitor.

^b^LMS: learning management system.

^c^SAP: sensor-augmented pump therapy.

**Figure 2:**
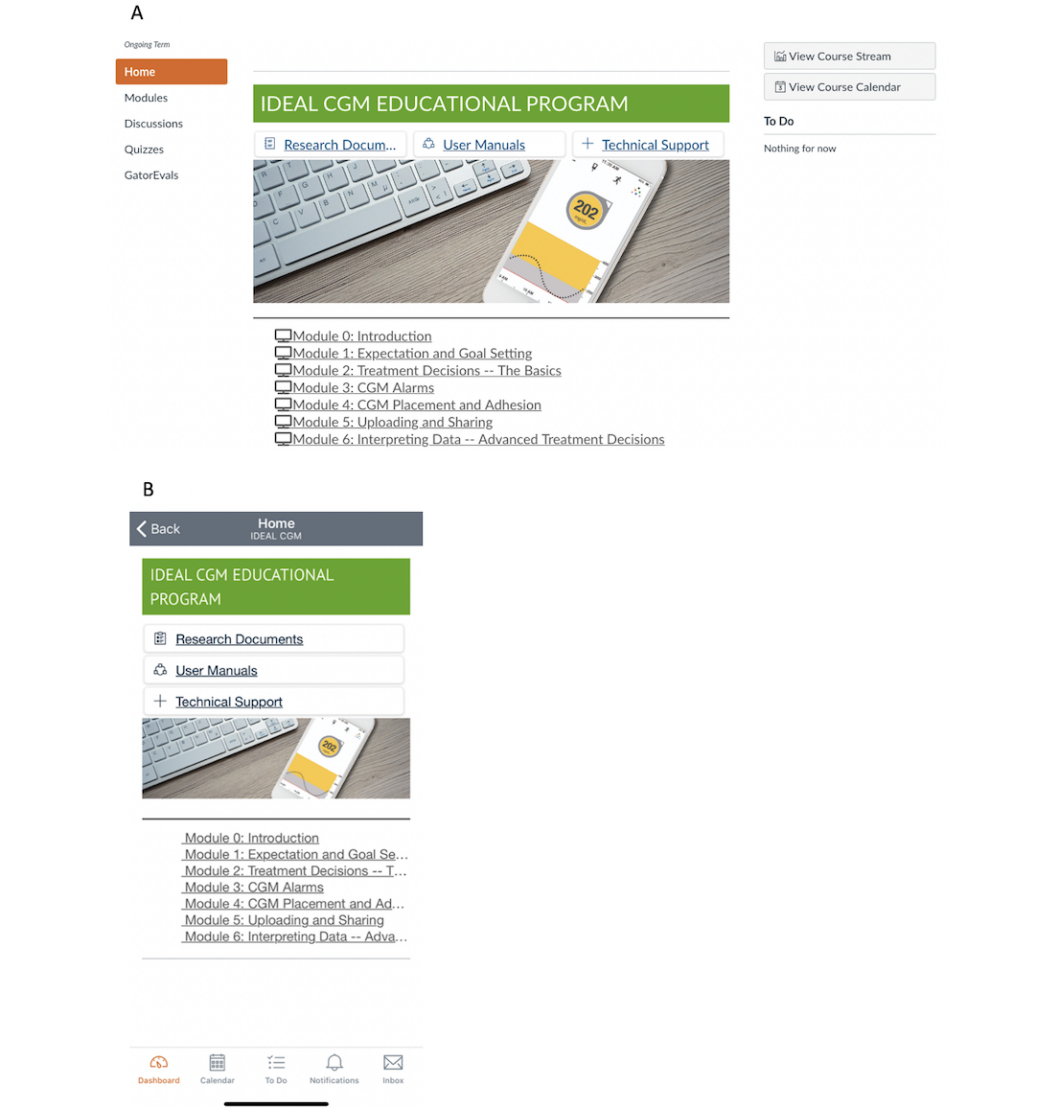
Screenshot of the IDEAL CGM (Intervention Designed to Educate and improve Adherence through Learning to use continuous glucose monitor) homepage. A. web-based and B. mobile-based.

The IDEAL CGM program was delivered via a learning management system that required a personal login and password to access via the desktop or mobile phone [43]. See Figure 2 for screenshots of the web-based and mobile-based home pages of the IDEAL CGM platform, which included access to asynchronous educational modules designed using professionally supported educational topics and training materials. Topics were created based on top patient-reported hassles leading to inconsistent or discontinued CGM use (ie, unmet expectations, alarm fatigue, placement/adhesion issues) [12], as well as training concepts pertinent to developing CGM self-efficacy and underscoring the benefits of use (ie, guidelines for treatment decisions, uploading/sharing data, and interpreting data; Multimedia Appendix 1). Peer-led discussion boards were linked to each module, which were intended to establish social support while facilitating peer-led observational learning. A health care professional monitored the discussion boards for appropriateness of content and provided tailored responses. Each module was designed using the same format and included a summary of the module topic, a “to-do” list with actionable items, a list of learning objectives, links to recorded video materials, additional materials to review, and recommended resources. Each week, proposed tasks included the review of recorded video materials, written educational content, and visual imagery, completion of the knowledge assessment checks, and participation within the peer-led discussion boards.

### Study Measures

We intended to examine the acceptability of the protocol, intervention dosage, participant responsiveness (user engagement in knowledge checks and discussion boards), and patient satisfaction with the IDEAL CGM program. Diabetes-related measures were described before and after the intervention and in relation to dosage of the intervention. Study data and survey responses were collected and managed using institutional review board–approved Research Electronic Data Capture (REDCap) tools hosted at the University of Florida [44]. REDCap is a secure, web-based app designed to support data capture for research studies. Electronic medical records and joint parent-youth interviews provided demographic and clinical data.

#### Feasibility Measures

##### Acceptability of the Protocol

Measures included recruitment and retention with a goal of at least 80% completion of baseline and follow-up measures.

##### Dosage and Participant Responsiveness

The learning management system collected and stored individual data related to dosage (ie, time spent, number of views, type of views) and participant responsiveness (ie, knowledge check submissions and discussion board posts) within the IDEAL CGM intervention.

##### Exit Satisfaction Survey

The exit satisfaction survey included 16 questions from the validated Flashlight Current Student Inventory, which was designed to gather information about a participant’s reaction to various teaching and learning practices [45]. The exit satisfaction survey used a 5-point Likert scale and open-ended questions to assess satisfaction related to the CGM training provided. Higher scores indicate more favorable satisfaction levels. The overall score is the mean of the item scores.

#### Diabetes-Related Measures

##### CGM Adherence

Usage data were collected by the CGM receiver and manually downloaded or automatically synced to a diabetes management platform. Adherence is described as the percentage of days that the CGM was worn over a 90-day period, with target adherence rates set to greater than 85%.

##### Glycemic Control

HbA_1c_ levels were measured using a DCA Vantage Analyzer (Siemens).

##### CGM Satisfaction

The CGM Satisfaction Scale [46], a 44-item validated measure, uses a 5-point Likert scale to assess satisfaction specific to CGM use and includes 2 subscales of “lack of hassles” and “benefits.” Higher scores indicate a more favorable impact and satisfaction with CGM use. Overall score is the mean of item scores.

##### CGM Self-efficacy

The CGM self-efficacy [16] version for youth older than 13 years, which is a 15-item validated measure, uses a 7-point Likert scale to assess the confidence of youth and parents to manage the technical and behavioral aspects of CGM use. Scores range from 0 to 100. CGM self-efficacy scores greater than 80 are considered “high” and are associated with adherence to CGM use and lower HbA_1c_ levels after 3 months [16]. The CGM self-efficacy survey has not yet been validated in youth 18 years or older.

##### Knowledge Assessment

The 20-question unvalidated assessment designed for the study used a multiple choice questionnaire to measure the attainment of knowledge related to the key aspects of CGM use. The knowledge assessment was scored as 0%-100%.

### Data Analysis

Intention-to-treat analysis was performed based on the randomization status of each participant. Participants randomized to the intervention group were included within analysis, regardless of the actual dosage or participant responsiveness within the intervention. Analysis was performed in SPSS (Version 25, IBM Corp). Descriptive statistics were presented for individual participant data with group median and range provided.

## Results

### Measures of Feasibility

#### Acceptability of the Protocol

**Figure 3:**
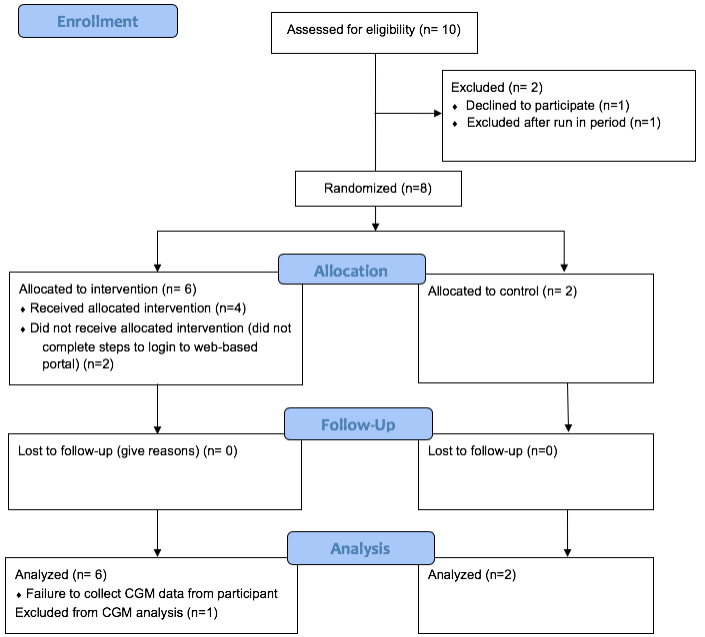
Study flow diagram. CGM: continuous glucose monitor.

The acceptability of the protocol is demonstrated by the study flow diagram (Figure 3). Of the 10 patients assessed for eligibility, 8 (80%) agreed to participate and were randomized to the enhanced standard care versus intervention plus enhanced standard care groups. For ease of interpreting study results, participants (P) were numbered 1-8 and were categorized based on intervention (i) or enhanced standard care/control group (c). P1-i through P6-i identify those randomized to the intervention, while P7-c and P8-c were randomized to the enhanced standard care group. The baseline and clinical characteristics of the 2 groups were comparable, as shown in Table 3.

This study demonstrated the ability to retain participants with a very low attrition rate. All survey measures were completed. Six of the 8 participants (75%) returned to clinic within the 3-month (SD, 2 weeks) study window for HbA_1c_ assessment, while the assessments for the other 2 participants (P1-i and P4-i) were performed outside of the intended window. CGM data were collected from 7 participants (88%) at follow-up. P1-i failed to bring the personal receiver in for upload and was unable to upload remotely.

**Table 3:** Baseline characteristics and clinical features of the enrolled participants.

Participant (P)		Age (years)	Sex	Race	Ethnicity	Current pump use	Previous CGM^a^ use
**Intervention (i) group**							
	P1-i	17	Male	White	Non-Hispanic	Yes	N/A^b^
	P2-i	16	Female	Mixed	Non-Hispanic	No	N/A
	P3-i	17	Male	White	Non-Hispanic	No	N/A
	P4-i	15	Female	White	Non-Hispanic	Yes	N/A
	P5-i	20	Female	White	Hispanic	No	Brand: Dexcom Duration of use: 2 weeks Date: 2 years prior
	P6-i	16	Male	White	Non-Hispanic	No	Brand: Dexcom Duration: 12 weeks Date: 6 months prior
**Enhanced standard care group or control (c) group**							
	P7-c	17	Female	White	Non-Hispanic	No	N/A
	P8-c	18	Male	Not reported	Hispanic	Yes	Brand: Medtronic Duration of use: 1 week Date: 4-5 years prior

^a^CGM: continuous glucose monitor.

^b^N/A: not applicable (they were naïve to CGM prior to study).

#### Dosage and Participant Responsiveness

**Figure 4:**
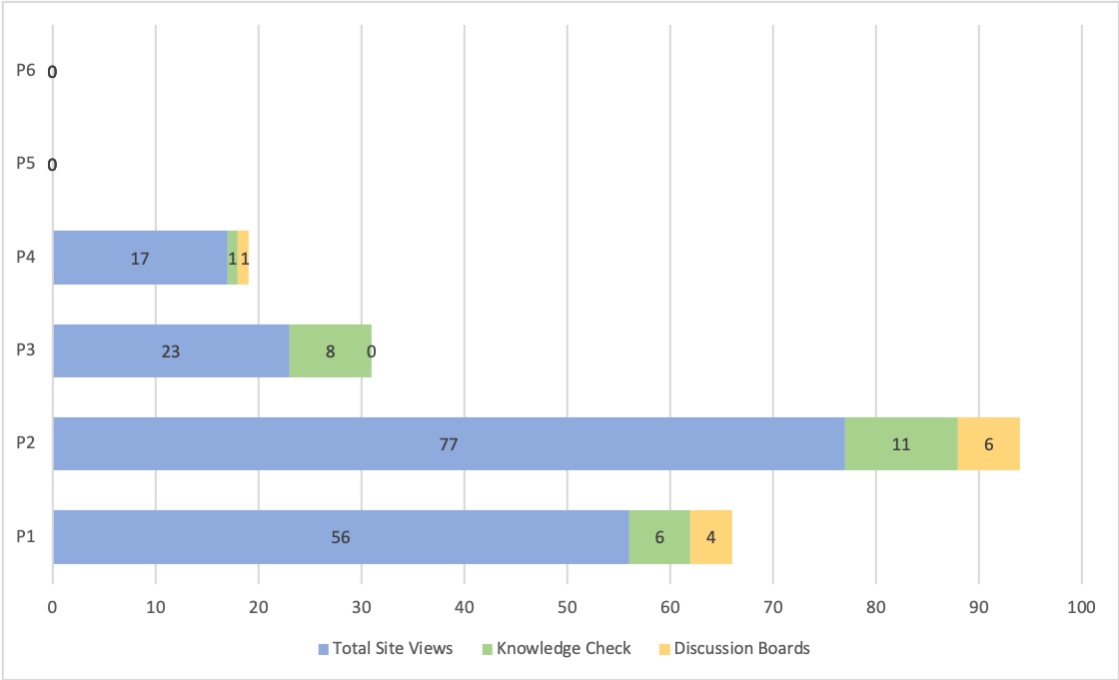
Overview of participant dosage and responsiveness within the intervention. P: participant.

The number of modules viewed by the participants varied widely. The overall average view rate of the modules was 48% (3.3/7 modules). In total, 4 of the 6 intervention participants completed the steps required to login to the IDEAL CGM program and view the training modules; the remaining 2 never logged into the intervention platform. Half of the intervention participants (n=3) were engaged in at least 5 of the 7 modules or more than 70% of the intended modules. However, the time spent within the modules and participant responsiveness varied. The median time spent within the web-based platform was 32 minutes (range 0-138 minutes). Figure 4 displays the dosage and type of engagement within the web-based intervention for each participant. P2-i and P3-i completed specific knowledge checks more than once (range 2-5 times). See Multimedia Appendix 2 for additional details regarding the frequency and type of participant engagement within each module.

#### Participant Satisfaction

Overall, participants within both groups reported being satisfied with their CGM training and perceived level of active and collaborative learning. Four participants within the intervention group indicated they were “very satisfied” with their CGM education, while 2 were “satisfied” (P4-i and P6-i). One participant within the standard care group reported being “very satisfied” while one reported being “satisfied.” Scores ranged from 3.3 to 4.4 within the intervention group and 2.9 to 3.0 within the enhanced standard care group.

When asked to describe what they liked most about the CGM training provided, participants from the intervention group reported “being able to relate to other peers,” “the people were relatable to my lifestyle and how to accommodate any problems I had,” and “they made it easy to understand and easy to use for me.” Only participants with exposure to the intervention included comments related to peer engagement and observational learning. When asked to describe what they disliked the most, participants from the intervention group reported the need for “more study reminders,” the use of “shorter videos,” and the need to “rewatch the videos.” A complete list of open-ended participant feedback regarding CGM training is included in Multimedia Appendix 3.

### Diabetes-Related Outcomes

Participant data are summarized in Table 4.

**Table 4:** Diabetes-related outcome measures at baseline and follow-up per participant.

Measures		P1-i^a^	P2-i^a^	P3-i^a^	P4-i^a^	P5-i^a^	P6-i^a^	P7-c^b^	P8-c^b^
**CGM^c^ adherence (%)**									
	3 months	—^d^	61	89	10	62	12	89	94
**Glycemic control (HbA_1c_%)**									
	Baseline	11.6	>14	12.3	10.2	8.5	>14	8.7	10.7
	Follow-up	9.8	>14	9.8	9	8.4	>14	9.3	9.5
**CGM satisfaction survey score (max score 5)**									
	Baseline	4.7	1.3	3.8	3.6	4.3	3.5	3.9	4.2
	Follow-up	3.9	4.0	3.9	3.8	4.3	3.6	3.9	3.9
**CGM self-efficacy survey score (max score 100)**									
	Baseline	100	100	94	83	97	68	93	96
	Follow-up	89	84	92	78	99	50	98	84
**CGM knowledge assessment score (max score 100)**									
	Baseline	40	65	80	65	70	40	60	85
	Follow-up	55	80	65	60	55	45	70	85

^a^Participant in the intervention group.

^b^Participant in the enhanced standard care group.

^c^CGM: continuous glucose monitor.

^d^Not available.

### CGM Adherence

CGM adherence was clustered around 3 levels of use for the intervention group (P1-i to P6-i). One participant reached recommended use of at least 85% (P3**-**i, 80/90 days, 89%); 2 participants fell just shy of recommendations with greater than 60% use (P2-i, 55/90 days, 61%; P5-i, 56/90 days, 62%), and 2 participants had less than 15% use (P4-i, 9/90 days, 10%; P6-i, 11/90 days, 12%). The 2 participants within the standard care group reached recommended use of at least 85% (P7-c, 80/90 days, 89%; P8-c, 85/90 days, 94%). No CGM adherence data were collected for participant P1-i.

### Glycemic Control

Four participants within the intervention group saw an improvement in HbA_1c_ levels, ranging from 0.1% to 2.5%. The remaining 2 participants randomized to the intervention arm (P2-i and P6-i) had an HbA_1c_ level of greater than 14% at baseline and follow-up; therefore, potential improvements could not to be detected using the point-of-care HbA_1c_ analyzers. Of the participants within the enhanced standard care group, P8-c saw a 1.2% improvement in HbA_1c_ levels, while P7-c saw a worsening in HbA_1c_ levels (8.7% increased to 9.3%) after 3 months of CGM use.

### Psychosocial Measures

Within the intervention group, median CGM satisfaction scale scores improved from 3.7 at baseline (range 1.3-4.7) to 3.9 at follow-up (range 3.6-4.3). Within the enhanced standard care group, P8-c described a –0.3 decline in satisfaction from 4.2 to 3.9 while the satisfaction of P7-c remained unchanged from baseline to follow up (3.9). Within the intervention group, the median CGM self-efficacy scores decreased from 96 at baseline (range 68-100) to 87 at follow-up (range 50-99). Within the enhanced standard care group, 1 participant (P7-c) showed an increase in the score while the other participant (P8-c) showed a decrease in the score. Despite decreases in the self-efficacy, follow-up CGM self-efficacy scores remained “high“ (greater than 80) for all except for the 2 participants with the lowest CGM adherence (9/90 days, 10% and 11/90 days, 12%) and limited to no engagement within the intervention (P4-i and P6-i) [16].

### Knowledge Assessment

Within the intervention group, median CGM knowledge assessment scores were 65 at baseline (range 40-80), which decreased to 58 at follow-up (range 45-80). CGM knowledge assessment scores widely varied from baseline to follow-up, with some participants demonstrating knowledge attainment while others showed worsened scores. The 2 participants with exposure to at least 6 of the intervention modules demonstrated the greatest improvements in CGM knowledge, with a 15-point increase in score.

## Discussion

### Principal Findings

This pilot study examined the feasibility of the IDEAL CGM intervention and described patient adherence to CGM, changes in glycemic control, psychosocial measures, and knowledge levels in the intervention and enhanced standard care groups. Initial findings from the pilot sample of 8 participants demonstrated proof of concept and provided key design considerations for future efforts aimed at utilizing web-based training interventions. Overall, patients were satisfied with the IDEAL CGM training intervention and perceived high levels of active and collaborative learning during CGM training. Open-ended responses suggested the impact of the peer-led discussions on perceived social support. Additional research is necessary to determine the feasibility of using web-based training to improve adherence to CGM in adolescents and young adults new to CGM use. The heterogeneity of this population suggests the vastly differing levels of training and follow-up support necessary to improve CGM adherence and help patients reach glycemic targets. Aside from training alone, this study demonstrates the importance of considering baseline characteristics, factors motivating CGM use, intervention participation, and the translation of knowledge into learned behaviors. While some participants reached clinically relevant improvements in HbA_1c_ levels and sustained CGM use following relatively minimal to moderate levels of personalized training and follow-up support, other participants were likely in need of additional resources to maximize these outcomes. Aside from behavior, confounding variables such as diabetes distress, family conflict, perceived support, and psychological barriers should be investigated when limited improvements in HbA_1c_ levels occur despite high CGM adherence.

### Limitations

Study recruitment and the potential to determine feasibility were limited by the Food and Drug Administration’s approval of an upgraded version of the Dexcom CGM (Dexcom G6) ahead of the expected timeline. Both providers and patients often opt to wait until the release of the newest CGM technology. When possible, future training interventions should create materials that remain relevant, despite updates within the technology, and should exist in a format that can be easily updated to keep up with the continuous evolution and development of diabetes technology. Further, as CGM use becomes the standard of care within T1D management, many patients are started on these systems soon after diagnosis. Historically, research protocols have excluded patients recently diagnosed within the last 6-12 months to account for confounding variables affecting improvements in glycemic control (ie, intensive insulin therapy and residual beta-cell function). However, this shift within the clinical paradigm will likely affect studies’ ability to recruit patients naïve to diabetes technologies 6-12 months past diagnosis.

### Conclusion

Web-based training and support interventions should continue to be explored for their potential to improve adherence and glycemic outcomes, while minimizing the burden or psychosocial impact of use during the uptake of new diabetes technologies. Web-based interventions increase patient exposure to diabetes-self management education with little to no added burden to the health care team. Continued efforts should work to establish evidence-based training standards and follow-up support methods necessary to achieve the diabetes-related outcomes associated with CGM use. Further research is needed to demonstrate the feasibility of using a web-based intervention to increase knowledge, maximize patient responsiveness, and ensure the successful uptake of and consistent use of CGM technology by adolescents and young adults.

## Appendix

Multimedia Appendix 1 Description of module topics within IDEAL CGM (Intervention Designed to Educate and Improve Adherence Through Learning to Use Continuous Glucose Monitor) training intervention.

Multimedia Appendix 2 Detailed view of participant dosage and responsiveness within the IDEAL CGM (Intervention Designed to Educate and Improve Adherence Through Learning to Use Continuous Glucose Monitor) training intervention.

Multimedia Appendix 3 Open-ended exit satisfaction survey responses from each participant.

Multimedia Appendix 4 CONSORT-eHEALTH checklist (V 1.6.1).

## Abbreviations

CGM: continuous glucose monitor
IDEAL: Intervention Designed to Educate and Improve Adherence Through Learning
HbA_1c_: hemoglobin A_1c_
REDCap: Research Electronic Data Capture
T1D: type 1 diabetes
