# Supplementary material for: Feasibility of the Web-Based Intervention Designed to Educate and Improve Adherence Through Learning to Use Continuous Glucose Monitor (IDEAL CGM) Training and Follow-Up Support Intervention: Randomized Controlled Pilot Study
Source: JMIR Diabetes. 2021 Feb 9;6(1):e15410. doi: 10.2196/15410 (PMC7902192; doi:10.2196/15410)
Supplement: Multimedia Appendix 1 [file diabetes_v6i1e15410_app1.docx]

**Appendix 1.** Description of module topics within IDEAL CGM training intervention.

| Module | Topic/ Description |
| --- | --- |
| Module 0 | Introduction/Icebreaker; Introduction to the program, review of expectations, and rules and regulations for Canvas discussions. |
| Module 1 | Expectations/Goal Setting; Address CGM expectations and benefits as well as known hassles. Develop personalized steps to achieving self-determined CGM goals. |
| Module 2 | Guidelines for Treatment Decisions; Review FDA approved guidelines for non-adjunctive CGM use. |
| Module 3 | Avoiding Alarm Fatigue; Address concept of alarm fatigue and recommendations for avoiding. Review alerts and alarm options. |
| Module 4 | CGM Placement and Adhesion/Skin Sensitivity; Tips/tricks to overcome difficulties regarding adhesion and skin sensitivities. Determining placement sites. Incorporated discussion of body image issues. |
| Module 5 | Uploading/Sharing Data; Learning tutorial for uploading data to the appropriate data management platform for personal and professional review. Review options and discuss personal preferences for sharing data. |
| Module 6 | Interpreting Data; Explanation and review of trend arrows. When to contact your diabetes care team for insulin adjustments. |

Abbreviations: CGM, continuous glucose monitor; FDA, Food and Drug Administration.
