# Supplementary figures and images for: Feasibility of the Web-Based Intervention Designed to Educate and Improve Adherence Through Learning to Use Continuous Glucose Monitor (IDEAL CGM) Training and Follow-Up Support Intervention: Randomized Controlled Pilot Study
Source: JMIR Diabetes. 2021 Feb 9;6(1):e15410. doi: 10.2196/15410 (PMC7902192; doi:10.2196/15410)

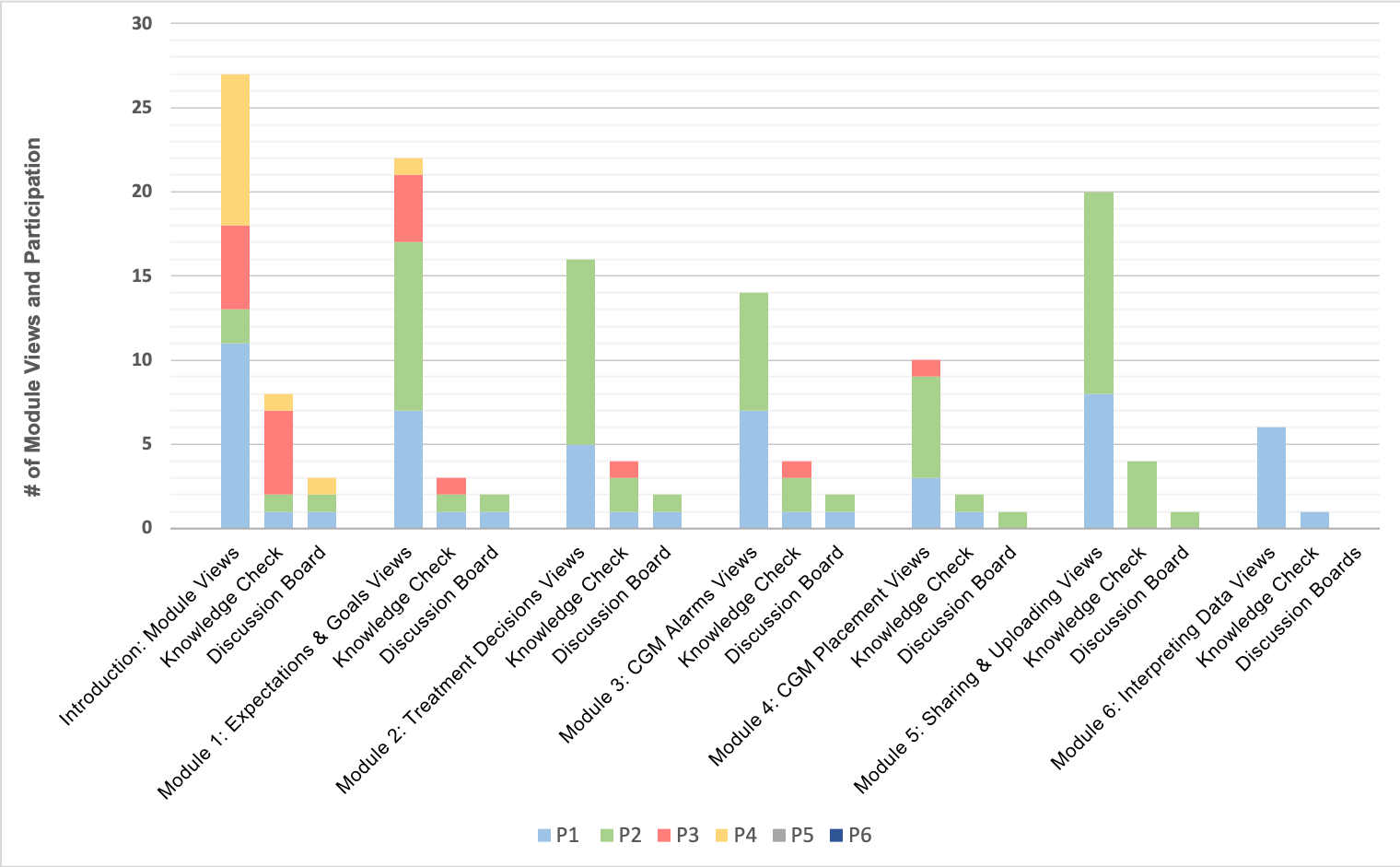

Supplement: Multimedia Appendix 2 [file diabetes_v6i1e15410_app2.png]
