# Supplementary material for: Feasibility of the Web-Based Intervention Designed to Educate and Improve Adherence Through Learning to Use Continuous Glucose Monitor (IDEAL CGM) Training and Follow-Up Support Intervention: Randomized Controlled Pilot Study
Source: JMIR Diabetes. 2021 Feb 9;6(1):e15410. doi: 10.2196/15410 (PMC7902192; doi:10.2196/15410)
Supplement: Multimedia Appendix 3 [file diabetes_v6i1e15410_app3.docx]

**Appendix 3.** Open-ended exit satisfaction survey responses from each participant.

|  | “What did you like the most about the training provided for learning to use CGM?” | “What did you dislike the most about the training provided for learning to use CGM?” | “What advice can you give us to improve the training provided for learning to use this technology?” |
| --- | --- | --- | --- |
| P1 -- i: | The new things I didn't know before. | I liked it all. | None, it's just right. |
| P2 -- i: | Being able to relate to other peers. | -- | Shorter videos. |
| P3 -- i: | They made it easy to understand and easy to use for me. | Nothing keep doing what you are doing. | Nothing. |
| P4 -- i: | The people were relatable to my life styles and how to accommodate any problems I had. | The first time I went to do training, it went by too fast. The second time was much better. | For the study, more reminders because I fell behind. |
| P5 -- i: | It gave detailed video instruction for the removal and application of the CGM and was easy to follow. | Nothing. | Everything was excellent in my opinion. |
| P6 -- i: | Help me know more about diabetes. | Nothing. | I don’t know |
| P7 -- c: | The pace of it. It was simple and easy. | Nothing. Overall was pretty good. | Let the patient do it theirselves a couple of times, because that's where you will get the most questions. |
| P8 -- c: | -- | -- | -- |

Abbreviations: c, standard clinical care/control group; CGM, continuous glucose monitor; i, intervention; P, participant.
